# Correction: Han et al. MicroRNA-146b-5p Suppresses Pro-Inflammatory Mediator Synthesis via Targeting TRAF6, IRAK1, and RELA in Lipopolysaccharide-Stimulated Human Dental Pulp Cells. *Int. J. Mol. Sci.* 2023, *24*, 7433

**DOI:** 10.3390/ijms25042049

**Published:** 2024-02-08

**Authors:** Peifeng Han, Keisuke Sunada-Nara, Nobuyuki Kawashima, Mayuko Fujii, Shihan Wang, Thoai Quoc Kieu, Ziniu Yu, Takashi Okiji

**Affiliations:** Department of Pulp Biology and Endodontics, Graduate School of Medical and Dental Sciences, Tokyo Medical and Dental University (TMDU), Tokyo 113-8549, Japan

In the original publication, there was a mistake in Figure 4J,K as published [1]. An incorrect DNA fragment was mistakenly used as 3′-UTR of RELA for the hsa-miR-146b-5p binding assay. To correct this mistake, the authors performed a luciferase analysis with the correct constructed DNA fragment for 3′-UTR of RELA. The corrected Figure 4J,K appears below. The authors state that the scientific conclusions are unaffected. This correction was approved by the Academic Editor. The original publication has also been updated.

**Figure 4:**
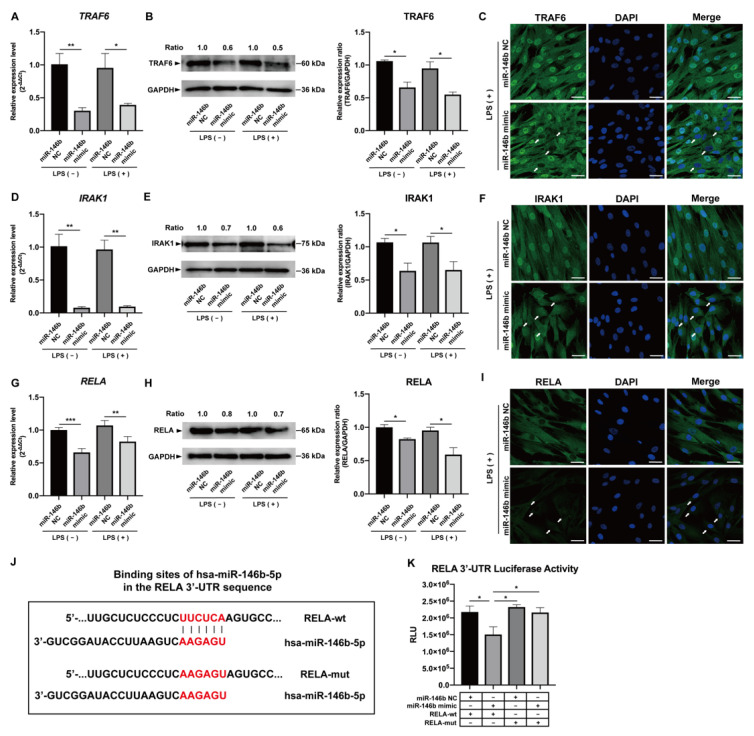
hsa-miR-146b-5p mimic down-regulates TRAF6, IRAK1, and RELA in LPS-stimulated hDPCs. hsa-miR-146b-5p mimic significantly down-regulated the mRNA (**A**,**D**,**G**) and protein (**B**,**C**,**E**,**F**,**H**,**I**) expression of TRAF6, IRAK1, and RELA in hDPCs under 2 h LPS stimulation (mean ± SD, n ≥ 3). (**J**) Wild-type (RELA-wt) and mutated (RELA-mut) target sequences of hsa-miR-146b-5p within RELA 3′-UTR are illustrated. (**K**) Luciferase reporter assay shows down-regulation of luciferase activity of RELA 3′-UTR in wild-type cells with over-expression of hsa-miR-146b-5p (mean ± SD, n = 4). * *p* < 0.05, ** *p* < 0.01, and *** *p* < 0.001. LPS: lipopolysaccharide; hDPCs: human dental pulp cells; miR-146b NC: miRNA mimic Negative Control #1; miR-146b mimic: miRNA mimic for hsa-miR-146b-5p; RLU: relative light unit; White arrows: target gene expression of TRAF6, IRAK1 or RELA was down-regulated in cytosol or nucleus in the white arrow indicated cells; Scale bars: 50 μm.
