# Supplementary material for: MicroRNA-146b-5p Suppresses Pro-Inflammatory Mediator Synthesis via Targeting TRAF6, IRAK1, and RELA in Lipopolysaccharide-Stimulated Human Dental Pulp Cells
Source: Int J Mol Sci. 2023 Apr 18;24(8):7433. doi: 10.3390/ijms24087433 (PMC10138803; doi:10.3390/ijms24087433)
Supplement: Supplementary file 1 [file ijms-24-07433-s001.zip › Figure S1.pdf]

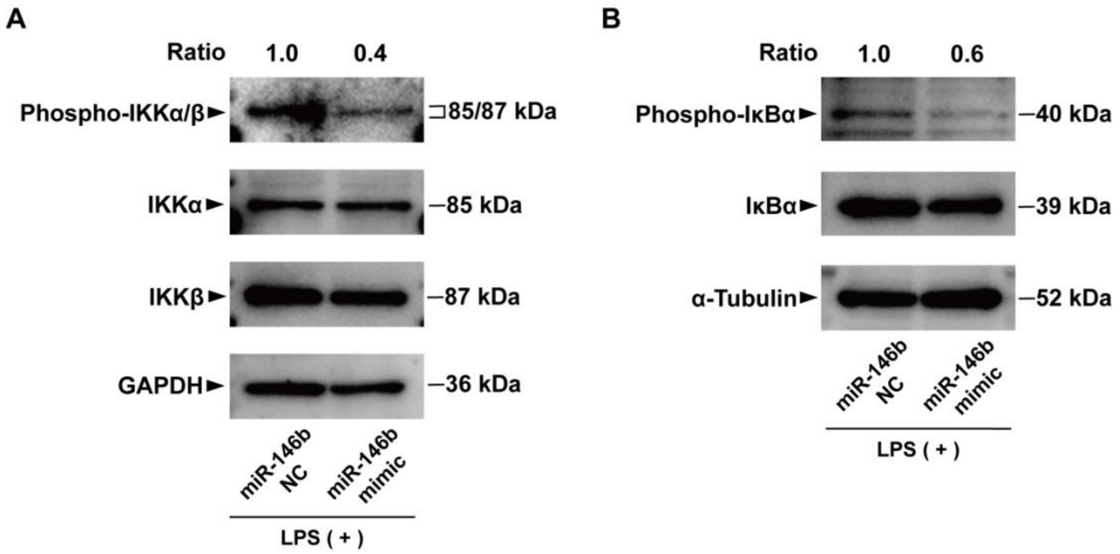

Figure S1. Phosphorylation of IKKα/β and IκBα was down-regulated by hsa-miR-146b-5p. (A) Expression of phosphorylated IKKα/β was down-regulated by hsa-miR-146b-5p mimic in LPS-stimulated hDPCs. (B) Expression of phosphorylated IκBα was down-regulated by hsa-miR-146b-5p mimic in LPS-stimulated hDPCs. LPS: lipopolysaccharide; hDPCs: human dental pulp cells; miR-146b NC: miRNA mimic Negative Control #1; miR-146b mimic: miRNA mimic for hsa-miR-146b-5p.
