# Supplementary material for: MicroRNA-146b-5p Suppresses Pro-Inflammatory Mediator Synthesis via Targeting TRAF6, IRAK1, and RELA in Lipopolysaccharide-Stimulated Human Dental Pulp Cells
Source: Int J Mol Sci. 2023 Apr 18;24(8):7433. doi: 10.3390/ijms24087433 (PMC10138803; doi:10.3390/ijms24087433)
Supplement: Supplementary file 1 [file ijms-24-07433-s001.zip › Figure S2.pdf]

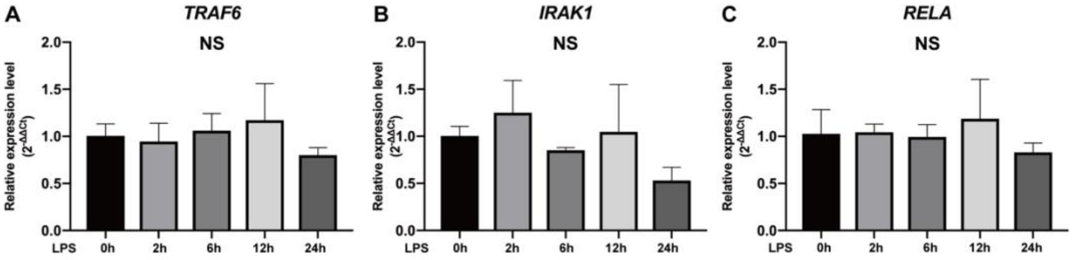

Figure S2. mRNA expression levels of *TRAF6*, *IRAK1*, and *RELA* in LPS-stimulated hDPCs at different time points. (**A**) mRNA expression of *TRAF6* was constant in LPS stimulation at different time courses (mean ± SD, n = 4). (**B**) mRNA expression of *IRAK1* was constant in LPS stimulation at different time courses (mean ± SD, n = 4). (**C**) mRNA expression of *RELA* was constant in LPS stimulation at different time courses (mean ± SD, n = 4). NS: no significant difference; LPS: lipopolysaccharide; hDPCs: human dental pulp cells.
